# Steam in Sewers

**Published:** 1886-04

**Authors:** 


### Steam in Sewers.

The question as to whether exhaust steam in sewers was injurious to the public health, was discussed at a recent meeting of the medical officers of our Municipal Board of Health. Dr. Geo. Fell, who has had an extensive experience as a civil and sanitary engineer, was invited to speak to the Board on this subject. In his opinion, the discharge of steam in sewers is not injurious to the public health, provided the sewers are ventilated. If, however, the sewers are not ventilated, the practice is dangerous, since by increasing pressure in the sewers, the sewer air might be forced through traps into dwelling-houses.

So great has been the reaction against the germ theories of disease in France since the publication of Gautier's articles, that

Gautier himself has requested the Savants of the Academy of Medicine not to be over hasty in making deductions from his discoveries. Some have gone so far as to insist that many, if not all, zymotic diseases may originate *de novo* in the body, without the intervention of any ferment from without.

---

The following formula is highly recommended by Dr. Duffy in the treatment of subacute rheumatism with anæmia, or the anæmia following acute rheumatism:

| | | |
|---|---|---|
| ℞ | Acid Salicylic, - - - | gr. viii. |
| | Sodii Salicylat, - - - - | gr. iv. |
| | Ferri Malat, - - - - | gr. i. |
| | Aq. - - - - - | ℥ ij. |

M. Sig.: One dose; to be taken every four hours.

---

Larger, who has recently been investigating the ætiology of *tetanus*, claims that it is never idiopathic, but always traumatic. Furthermore, he claims that it is always due to infection or contagion. From numerous observations of epidemics of tetanus, is seems probable that the contagion is not carried through the air, but is propagated through the medium of the soil.

---

There are now enrolled, as members of the night medical service of Paris, 608 physicians and 356 midwives, serving from 10 P. M. to 7 A. M. During the year 1885, 7,494 calls were received from a population of 2,226,000 persons. Of these calls, 52 per cent. were to attend women, 32 per cent. to attend men, and 16 per cent. to attend children.—*Progres Med.*

---

There is evidently a decline in the consumption of alcoholic liquors in this country at the present time. It is shown by the decrease of the internal revenue receipts last year, amounting to $9,000,000.

The present cheapness of quinine is in part due to the discovery of a quinine yielding bark in a plant which is not one of the cinchonas. *Cuprea* bark is easily cultivated in warm regions near to sea coast. This bark has less quinine in it than the cinchonas, but it is more easily extracted. Large quantities are raised in India and sent to the London market.

---

Who will now say that Buffalo is not a medical center? Four medical journals are now published here. We have two medical colleges, where twenty-two professors and twenty-one lecturers dispense their medical lore. Besides the regular city and county societies, there are now four private medical clubs, not including the homœopathic organizations.

---

According to a report made by the Illinois State Board of Health, there are in the United States and Canada 128 medical colleges. Of these, 36 require an attendance upon three courses of lectures, 110 advertise some sort of education before matriculation, 91 give instruction in hygiene, and 97 teach medical jurisprudence.

---

A fund is being raised in Paris, under the auspices of M. Verneuil, the surgeon, to encourage experiments for the cure of tuberculosis. Cornil and Bouchard will be on the list of investigators.

---

There are, in Italy, 18,000 physicians, about one to every 1,600 inhabitants. There are also 11,000 midwives, 2,800 "bleeders," and 235 dentists.

---

Jules Guerin, the eminent orthopædic surgeon of Paris, died January 27, 1886, aged 86 years.

Cholera is now prevalent in Brittany, France, the nearest point to America yet reached. There have been about twenty deaths this winter.

